# Assessment of eye health programme reach by comparison with rapid assessment of avoidable blindness (RAAB) survey data, Talagang, Pakistan

**DOI:** 10.1186/s12875-024-02503-4

**Published:** 2024-07-10

**Authors:** Muhammad Zahid Jadoon, Zahid Awan, Muhammad Moin, Rizwan Younas, Sergio Latorre-Arteaga, Elanor Watts, Marzieh Katibeh, Andrew Bastawrous

**Affiliations:** 1 Pakistan Institute of Community Ophthalmology, Peshawar, Pakistan; 2 CBM International, Islamabad, Pakistan; 3 College of Ophthalmology and Allied Vision Sciences, Lahore, Pakistan; 4 Peek Vision, Berkhamsted, UK; 5 Public Health Research Group, University of Alicante, Alicante, Spain; 6 Tennent Institute of Ophthalmology, Glasgow, UK; 7 Department of Ophthalmology, Faculty of Medicine, Aalborg University Hospital, Aalborg, Denmark; 8 International Centre for Eye Health, London School of Hygiene and Tropical Medicine, London, UK

**Keywords:** community health worker, primary eye care, eye health, survey, programme, epidemiology, cataract, refractive error, global ophthalmology

## Abstract

**Background:**

The purpose of this study was to quantify how much of the burden of visual impairment (VI) and unmet need in Talagang, identified by Rapid Assessment of Avoidable Blindness (RAAB) survey data, has been addressed by Community Eye Health (CEH) programme efforts.

**Methods:**

A RAAB survey was carried out in November 2018, with 2,824 participants in Talagang Tehsil, Punjab, Pakistan, aged 50 and over. Census data were used to extrapolate survey data to the population. Alongside this, a CEH programme was launched, consisting of community eye screening, and onward referral to rural health centres, secondary or tertiary ophthalmological services, as required. This health intervention aimed to address the eye care needs surfaced by the initial survey. From 2018 to 2022, 30,383 people aged 50 or over were screened; 14,054 needed referral to further steps of the treatment pathway and more detailed data collection. Programme data were compared to estimates of population unmet needs. Main outcome measures were prevalence of VI, and proportion of need met by CEH Programme, by cause and level of VI.

**Results:**

Among those aged 50 and over, 51.0% had VI in at least one eye. The leading causes were cataract (46.2%) and uncorrected refractive error (URE) (25.0%). In its first four years, the programme reached an estimated 18.3% of the unmet need from cataract, and 21.1% of URE, equally in both men and women.

**Conclusions:**

Robustly collected survey and programme data can improve eye health planning, monitoring and evaluation, address inequities, and quantify the resources required for improving eye health. This study quantifies the time required to reach eye health needs at the community level.

## Background

Globally, in 2020, it was estimated that 43.3 million people were blind (presenting visual acuity, VA, worse than 3/60 in the better eye), with nearly half of these cases due to cataract (17.0 million) or uncorrected refractive error (URE, 3.7 million) [1]. Cataract and URE combined are also responsible for 241.2 million of the 295.1 million people with moderate-severe visual impairment (VI). In addition, at least 509.7 million people are estimated to have near vision impairment due to uncorrected presbyopia. Cataract surgery and refractive correction are relatively simple interventions which could restore good vision to those affected. Provision of these services can be monitored via indicators including prevalence of VI, effective cataract surgical coverage (eCSC) and effective refractive error coverage (eREC) [2]. eCSC and eREC have been recommended by a WHO-led expert panel for assessment of eye care provision and Universal Health Coverage [3]. Standardised reporting allows consistent situational assessment, service planning, and comparison of groups and regions.

Relevant data is collected within the Global Vision Database/Vision Atlas [4], a key source of which is Rapid Assessment of Avoidable Blindness (RAAB) surveys [5, 6]. RAAB surveys assess prevalence and causes of VI in people aged 50 and over, as well as estimating cataract surgical coverage and identifying barriers to cataract treatment, and have been undertaken in over 80 countries. The over 50s age group is prioritised for screening when targeting those with cataract and vision impairment, as approximately 73% of blindness and visual impairment cases are in this group, with cataract being the leading cause [7].

Sequential RAAB surveys have been used to analyse service provision [8], and RAAB data has elsewhere been combined with Ministry of Health data regarding surgeries performed to allow review of eCSC [9].

Community-based eye care programmes can improve access to eye care, especially among low-income and rural patient groups [10]. Community eye health (CEH) programmes have been carried out in Pakistan with Peek Vision, who have previously demonstrated the use of appropriate data management and iterative programme change to allow for continuous programme improvement, including improved adherence to referral [11]. Comparison of data collected during these large-scale vision programmes and by RAAB surveys allows identification of groups which have been successfully reached by the programme, or missed, as well as projection of how much programme activity would be required to eradicate avoidable blindness and visual impairment in the region. Here we compare RAAB survey data from Talagang, Pakistan, with CEH programme data, to assess programme reach.

## Methods

### Visual impairment definitions

VI was defined as follows: early/mild: distance visual acuity (DVA) worse than 20/40 (6/12) but equal to or better than 20/60 (6/18); moderate VI: <20/60 (6/18), ≥ 20/200 (6/60); severe VI: <20/200 (6/60), ≥ 20/400 (3/60); and blindness: <20/400 (3/60), as per WHO/ICD-11 definitions. Moderate-severe VI (MSVI) therefore describes VA < 20/60 (6/18), ≥ 20/400 (3/60). As VA was measured monocularly, VI was assessed on a per-eye level, i.e. proportion of eyes with VI, and on a person level, based on VA in the better eye.

### RAAB survey

During November and December 2018, a RAAB survey was carried out in the Talagang Tehsil, Chakwal District, Punjab, Pakistan. As per standard RAAB methodology, clusters were randomly and systematically identified, and then 50 people aged 50 or over were selected from within each cluster via compact segment sampling. This led to an initial sample of 2,889 people. Data were collected via door-to-door fieldwork at participants’ homes. Assessment included VA measurement via Tumbling E chart, and examination with torch and direct ophthalmoscope. Pinhole vision testing and ophthalmologist examination were carried out for those with VA < 6/12. Dilated examination was carried out in those for whom a cause of visual impairment could not be found on undilated examination. This allowed estimation of prevalence of blindness and VI, and causes. Data included refractive error and presbyopia prevalence, spectacle wear coverage, as well as cataract surgical coverage (CSC), place of surgery, and barriers to cataract surgery.

### CEH programme

Alongside the RAAB survey, a CEH programme was launched in November 2018 in the same area, with collaboration between College of Ophthalmology and Allied Vision Sciences (COAVS), Peek Vision, and Christian Blind Mission (CBM). Communities were first sensitised by social organisers and Lady Health Visitors. Community eye screening was then carried out in Basic Health Units. Where indicated, screening participants were referred to rural health centres for refraction and provision of primary eye care. People with conditions requiring ophthalmological input were referred onwards to secondary or tertiary ophthalmological services. Hospitals were informed of referrals, and patients were sent automated text or voice messages reminding them of their appointments. The Peek software system was used to track this data, including the number of people screened, the screening quality, number of people referred, treated and non-attenders/left-behind groups. This allowed continuous improvement of the programme to improve uptake with a focus on equity and efficient use of the available resources. A more detailed summary of the programme has previously been described elsewhere [11].

### Census data

Since the RAAB and CEH programmes were carried out, 2017 census data [12] has been made available regarding the Talagang population. This has been used in this analysis for extrapolation to regional magnitude of visual impairment and blindness.

### Data security

The storage and transmission of this data was carried out in line with a Data Protection Agreement with the local stakeholders, and followed the European Union General Data Protection Regulation (GDPR).

### Data analysis

RAAB7 software and Peek Capture smartphone application were used to collect data for the survey and community programmes respectively. Data from RAAB survey automated reports were obtained from the RAAB repository with permission of the PI of the project (COAVS, Lahore). Population age and gender composition of people living in Talagang Tehsil were obtained from 2017 census data and used to extrapolate RAAB sample results to the population who live in the survey area. Anonymised programme data and RAAB data were processed using Stata 14.0 (StataCorp, Texas, US), R studio (R Studio, Massachusetts, US) and Google Sheets (Google, California, US). R Shiny software (R Studio, Massachusetts, US) was used for further data management and figure creation.

## Results

### Magnitude of visual impairment: RAAB Survey

**Fig. 1:**
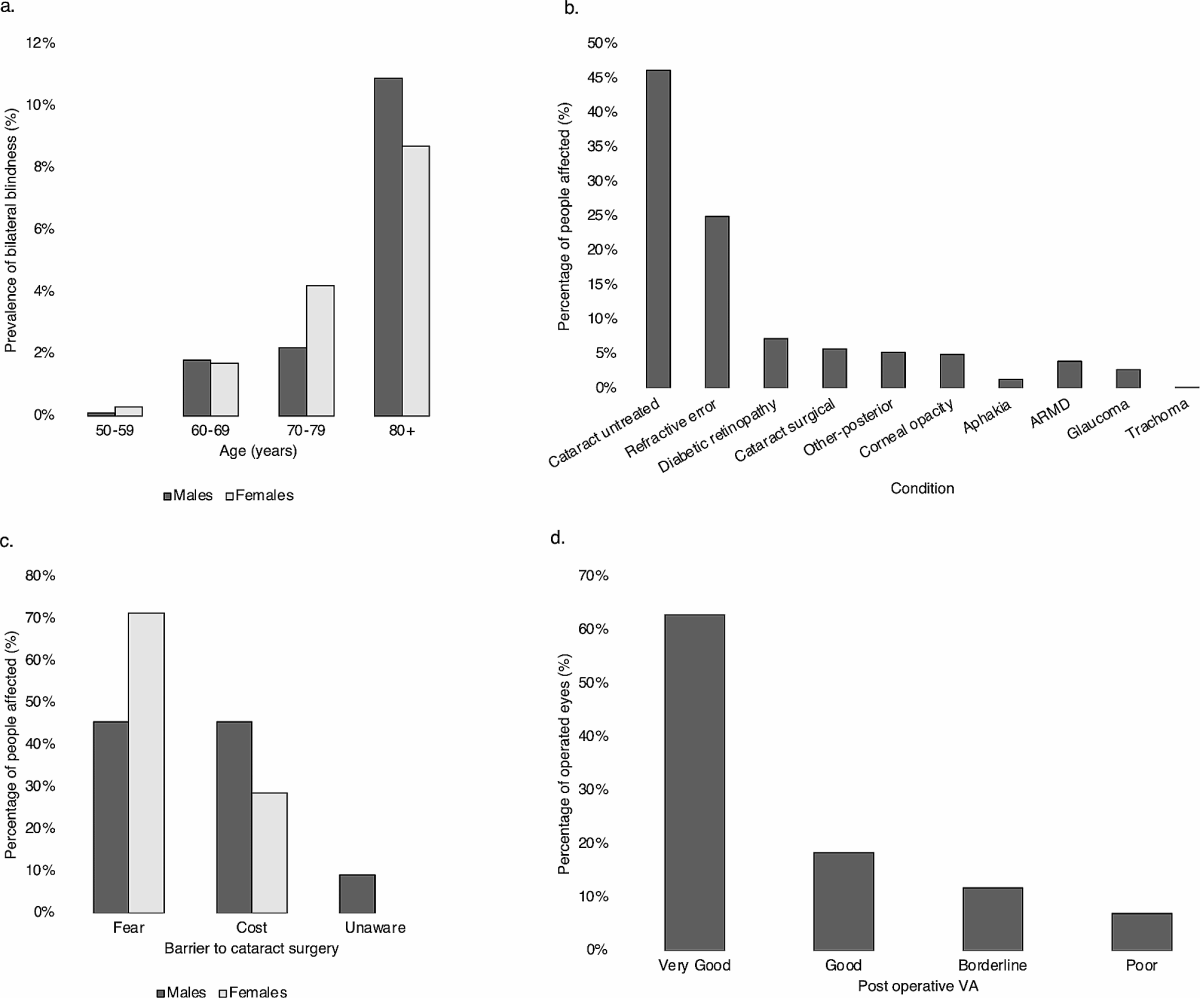
Summary of RAAB data. (**a**) Prevalence of bilateral blindness - best corrected visual acuity (BCVA) < 3/60 in better eye by age and gender. (**b**) Causes of blindness and severe or moderate visual impairment in the better eye (BCVA < 20/60 (6/18)). (**c**) Barriers to cataract surgery in people with bilateral BCVA < 6/60 due to cataract, by gender. (**d**) Outcomes of cataract surgery: BCVA in operated eyes. Very good: BCVA ≥ 6/12, Good: 6/12 > BCVA ≥ 6/18, Borderline: 6/18 > BCVA ≥ 6/60, Poor: BCVA < 6/60

Of 2,889 people who were invited to participate, 2,824 (response rate = 97.8%) participated in the RAAB survey. A summary of the results of the survey is presented in Fig. 1.

The sample prevalences of blindness, severe VI, moderate VI and early VI were 2.4% (95% CI: 1.6–3.3), 1.1% (95% CI: 0.7–1.5), 14.3% (95% CI:12.2–16.4), and 18.9% (95% CI: 15.3–22.5) respectively. In general, 42.8% of examined eyes (95% CI:39.6–46.0) and 36.7% of people (95% CI: 33.9–39.6), based on VA in the better eye, had any VI or blindness i.e. VA < 20/40 (6/12) with available correction.

Causes of blindness were avoidable in 97.6% of patients; the most common causes of blindness and visual impairment were: untreated cataract, uncorrected refractive errors, diabetic retinopathy, glaucoma, posterior segment pathology and corneal opacities. In participants with untreated cataract, the main barriers to uptake of cataract surgery were fear (55.6%) and cost (38.9%). In those who had had cataract surgery, the outcome was good (18.4%) or very good (62.8%) in 81.3% of cases.

### Implemented CEH programme

**Fig. 2:**
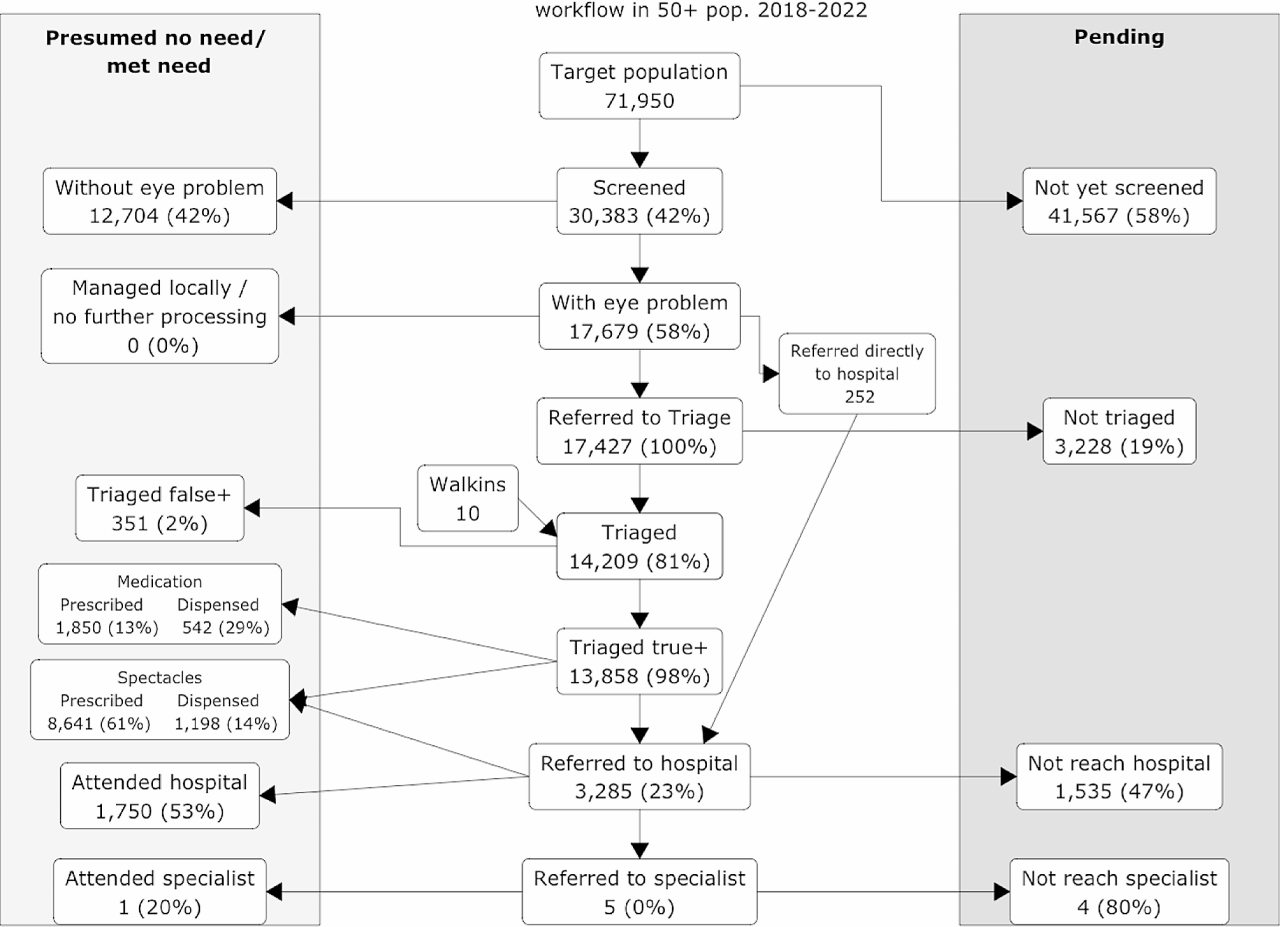
Programme workflow, November 2018 to November 2022, for the population aged ≥ 50 in Talagang Tehsil. The sum of the percentages of patients who have received spectacles, medication, hospital (ophthalmologist) or specialist referrals may exceed 100% as some patients have multiple outcomes

In Talagang, in the Community Eye Health (CEH) and School Eye Health programmes combined, between 2018 and 2022: 420,147 people were screened; 65,874 referred on to triage for further assessment; 31,454 people were prescribed spectacles, and 3,482 attended hospital services. Figure 2 shows an overview of the programme participants aged 50 or over, seen during the first four years of the Community Eye Health programme.

The eye health need surfaced by the RAAB survey and the amount of need that has been reached by the programme are compared in Table 1. Extrapolation of RAAB data estimates that 54.5% (39,233/71,950) of the 50 + population in Talagang had VI in at least one eye. 41.8% (30,090/71,950) of people had VI in their better seeing eye.

**Table 1:** Comparison of magnitude of visual impairment frequency between the RAAB sample and the CEH programme

	RAAB Sample			Extrapolation to Area Population (Adjusted for population demographics)			Eye Care Programme* (First four years)	
Presenting Visual Status in the Worse Eye (Uni- and Bilateral Visual Impairment)								
	Freq.	Percent		Freq.	Percent		Freq.	Percent
No VI	1445	51.1%		32,707	45.5%		6257	44.5%
Early VI	486	17.2%		12,064	16.8%		2187	15.6%
Moderate VI	552	19.5%		15,493	21.5%		3474	24.7%
Severe VI	123	4.4%		3742	5.2%		634	4.5%
Blindness	220	7.8%		7934	11.0%		1502	10.7%
Subtotal: any VI	1381	48.9%		39,233	54.5%		7797	55.5%
Presenting Visual Status in the Better Eye (Bilateral Visual Impairment)								
	Freq.	Percent		Freq.	Percent		Freq.	Percent
No VI	1788	63.3%		41,852	58.2%		8412	59.9%
Early VI	533	18.9%		14,001	19.5%		2293	16.3%
Moderate VI	404	14.3%		12,518	17.4%		2513	17.9%
Severe VI	32	1.1%		1016	1.4%		323	2.3%
Blindness	69	2.4%		2556	3.6%		513	3.7%
Subtotal: any VI	1038	36.7%		30,090	41.8%		5642	40.2%

*The programme data presented in this column is based on full visual acuity assessments in the triage centres. A further 12,704 people were identified as healthy during the screening programme using a pass/fail screening threshold of 20/40 (6/12).

**Fig. 3:**
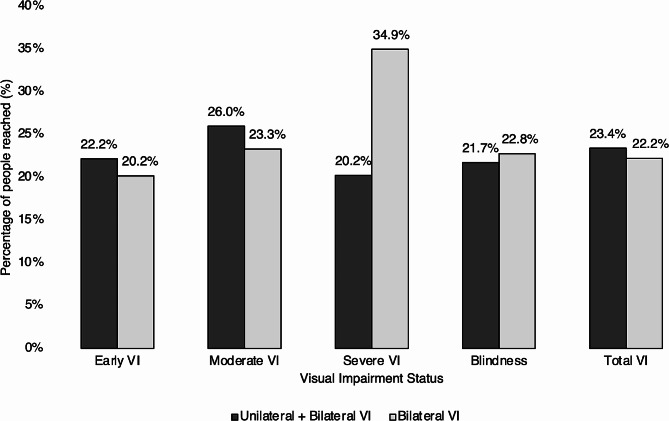
Combined reach to people with visual impairment in Talagang Tehsil, District Chakwal, Pakistan. Percentage of estimated unmet need reached during the RAAB survey and the first four years of the programme

As shown in Table 1, there were an estimated 39,233 people who had any VI or blindness in at least one eye at the beginning of the programme. The RAAB survey and CEH programme combined screened 9,178 (1,381 + 7,797) of those people (23.4%) during the first four years. Figure 3 shows the proportion of VI which was reached by the programme, by visual acuity status. In most groups, the proportion of reach was between 20 and 26%, while the programme reached over a third of people with bilateral severe VI.

**Fig. 4:**
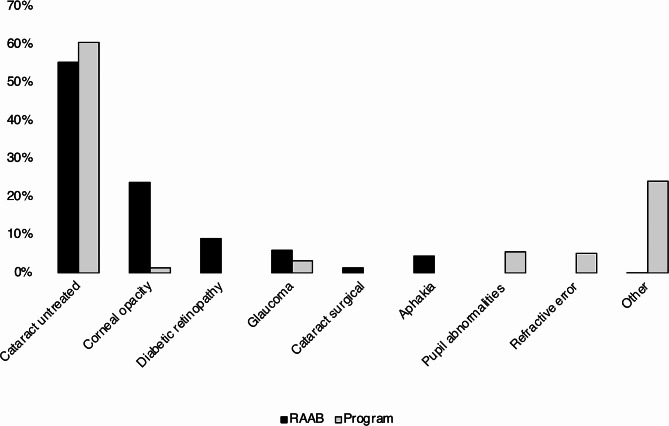
Causes of blindness in RAAB survey, 2018, and among CEH programme participants (triage stage). Programme data refers to the first four years of the programme

Figure 4 visualises the causes of blindness identified by RAAB survey and the distribution of eye conditions among the 513 blind people who were reached by programme. As shown in this figure, untreated cataract was the leading cause of blindness in 55% of cases in the survey and 61% of cases in the programme. URE was the second most common cause of VI (25.0%) but not a major cause of blindness.

**Fig. 5:**
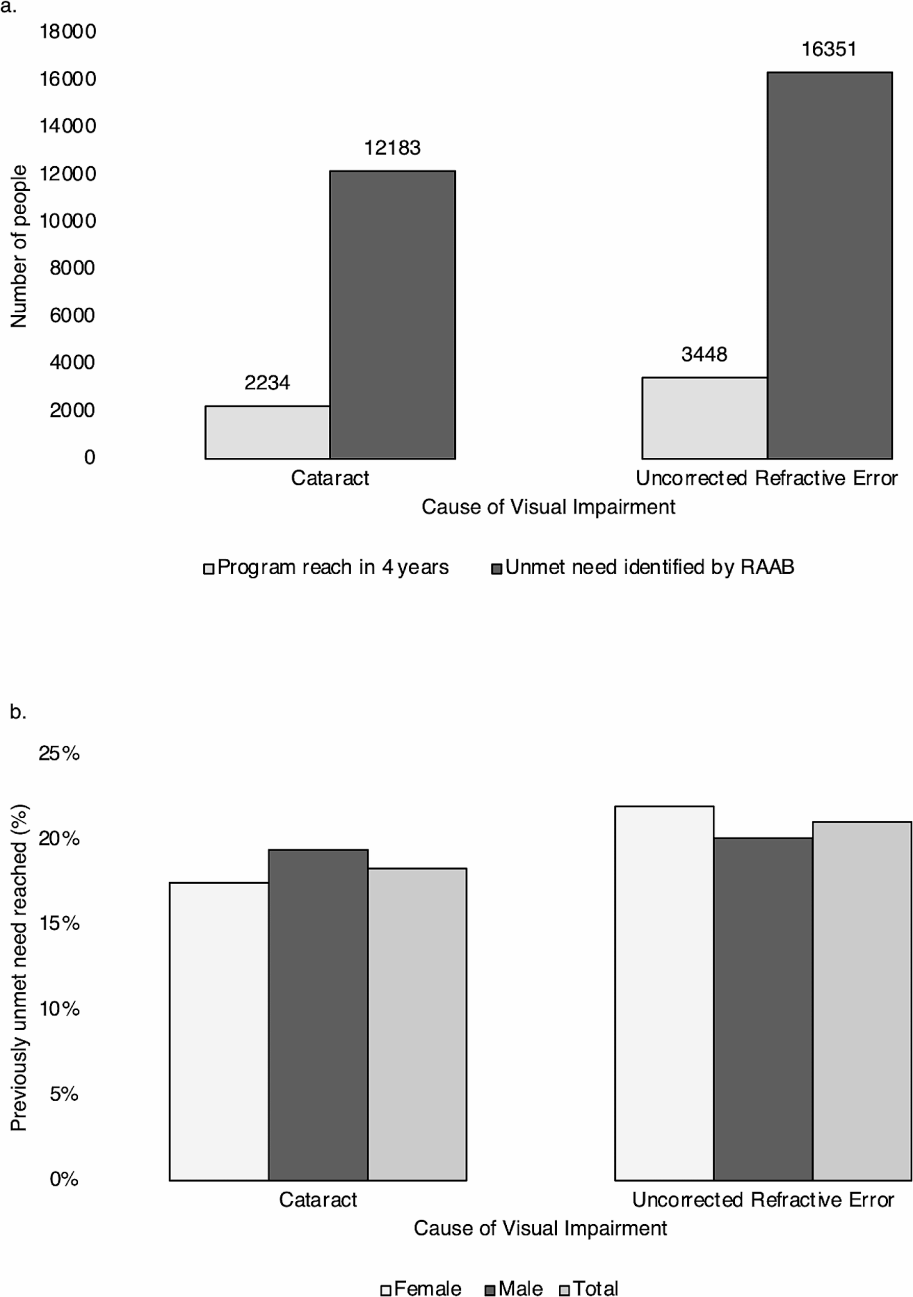
Cataract surgery and refractive service need in Talagang. (**a**) Magnitude of need identified by RAAB survey and reached by CEH Programme: Main causes of VI in either eye, (**b**) Percentage of previously unmet need reached during the first four years of the programme, by gender

As shown in Fig. 4, while cataract was the leading cause of blindness in both settings, the distribution of other causes differed between the programme and RAAB survey. The proportion of VI secondary to cataract or URE met by the programme is shown in Fig. 5: approximately 18.3% of cataract surgical need and 21.1% of need for refractive services in this area have been reached during the first 4 years of the programme (average 5% per year).

## Discussion

South Asia has both the highest age-standardised prevalence of moderate-severe VI of any Global Burden of Disease region, and the largest absolute number of cases [1]. Effective refractive error coverage (eREC) describes the proportion of people with vision impairing refractive error or presbyopia, whose need has been sufficiently met, i.e. with refractive correction (spectacles or contact lenses) that resolves their vision impairment. There is vast global inequity in eREC, with 79.1% distance eREC in high-income countries, but only 9.0% in South Asia and 5.7% in Sub-Saharan Africa [6]. Despite South Asia bearing such a large proportion of VI need, it is underrepresented in research into eye health service improvement: for example, in a recent global scoping review, it was found that the majority of research into interventions to improve cataract services has been undertaken in high-income countries, with only 6.3% in South Asia (*n* = 9/143) [13]. Encouragingly, there has been significant improvement of eREC in the region over the last two decades [6].

Robustly collected survey data can be used to improve CEH programmes at all stages of the programme. Surveys carried out prior to programme initiation allow for better understanding of which conditions are likely to be seen in a region, and their magnitude. In this way, RAAB surveys can be used to help plan service provision [14, 15]. Comparison of repeated eye health surveys (undertaken before and after CEH programme interventions) has enabled assessment of the effectiveness of the programmes carried out in the intervening period, for example in Nigeria [16] and India [17].

We presented an evidence-based and comparable estimation of the magnitude of eye care needs and causes of vision impairment in a district level population, applying RAAB methodology. This allowed the CEH programme to establish a defined baseline against which to measure its progress towards eradicating avoidable blindness. After introduction of RAAB methodology in 2006, over 300 RAAB studies have been completed in low- and middle-income countries. Previous studies have assessed sequential RAABs alongside health systems/workforce programme data [16], and compared RAAB results with historical cataract surgical rate (CSR) programme data [18]. However, to the best of our knowledge, this is the first study that compares baseline RAAB data with subsequent programme reach to demonstrate the need met by an eye health programme in a defined population, and that which still needs to be reached.

Based on initial RAAB data, this programme reached almost 20% of previously unmet need due to the main causes of visual impairment, equally in both men and women. Regional variation is large; a previously published RAAB in Pakistan, carried out in Lahore, found VI prevalence to be remarkably low in the area: 1.9%, with a CSC of 84.0%[19]. However, even with such good service coverage, there was significant inequity between genders, with CSC of 94.1% for men, and 72.1% for women. Here, VI prevalence was found to be much higher in Talagang. The proportion of cataract and URE need reached by this programme was equitable between men and women, as shown in Fig. 5b.

Census data is usually required for extrapolation from survey results to national or regional magnitude. In this case, Pakistan’s census had been delayed, such that at the initial time of RAAB survey, the most recent formal national census with available data was from 1998. As the 2017 census data are now available, the estimated need is higher than it was initially. This reduces the estimated proportion of need which has been met by the programme, though they remain encouraging at 18.3% of cataract and 21.1% of URE (see Fig. 5).

Since the time of data extraction and analysis, more people may have attended hospital eye services and more of the pending need may have been met. Regardless, focusing on strengthening the last part of the referral system, i.e. hospital attendance following referral (Fig. 2), seems an area with room for further improvement in this context, to close the loop of eye care needs. This was flagged as a priority by ongoing monitoring and evaluation during the programme, and work has already been undertaken to tackle this [11]. As a newly implemented programme, approximately one fifth of people in need have been successfully reached so far, which is very encouraging for this early implementation phase.

The leading cause of blindness as demonstrated in Fig. 4 was untreated cataract both in survey and programme data (55% and 61%). There were however discrepancies between other common causes, especially corneal opacities, which were reported with much higher frequency during the RAAB survey than in the CEH programme. Of note, more details of diagnosis were provided in the RAAB, while in programme data more cases were categorised as “other”. Corneal causes of VI may have been reported as “other” in some cases, leading to this difference. Although epidemiological data collection is not the primary goal of a programme, this might imply potential for improvement in precision of data collection within the programme, and could represent a limitation in our results. Alternatively, as RAAB surveys are statistically powered for their primary outcome: prevalence of blindness, the RAAB estimates of distribution of causes may be less precise.

Study limitations include that our estimates of need were based on point prevalence estimates from the RAAB survey, without modelling of new case incidence or population growth during the study time frame, or reduction in cases due to death or treatment elsewhere. This avoided introduction of an extra layer of uncertainty within our estimates, but means that some new cases may not be included in the estimates.

Another interesting result to note is that the prevalences of visual impairment and blindness were similar in the RAAB results to the group which was screened as “not healthy” in the first stage of the programme (Table 1), and attended triage. There are various possible explanations for this. If screening is accurate, with minimal false negatives, then disproportionately more healthy people without visual impairment are attending for screening than people with visual impairment. Screening out many of the healthy people is then returning proportions to the population baseline. This could occur if it is easier for people with good vision to become aware of the programme or to attend (e.g. due to ease of travel). Focusing on community awareness and programme enrolment methods such that people with visual impairment are more likely to participate could increase the effectiveness of the programme in reaching the population in need.

Further research is ongoing in the form of data collection within the continued programme. Visual outcome of patients who have undergone cataract surgery or received refractive correction would be beneficial to allow analysis of the effectiveness of interventions: eREC and eCSC. A repeat RAAB later would add helpful information regarding the change in prevalence of blindness and VI by each cause, following intervention by the programme.

## Conclusions

In this study, we compared start line RAAB survey estimates directly to programme data, to assess programme reach. The programme reached an estimated 20.1% of all expected cases of blindness in the survey area (*n* = 513/2,556) in the first four years, 18.3% of the unmet need from cataract, and 21.1% of URE, equally in both men and women. In combination, the survey and programme reached 23.4% of people with VI. Use of survey data in this way allows for improved monitoring and evaluation, highlighting any inequities in who is accessing the programme, as well as enabling calculation of the proportion of need which has been met. This can help planners of eye care programmes to allocate resources and to estimate the required duration of a programme to meet existing backlogs.

## Data Availability

The data used during the current study may be available from the corresponding author on reasonable request.

## Abbreviations

CEH: Community Eye Health
COAVS: College of Ophthalmology and Allied Vision Sciences
CSC: cataract surgical coverage
DVA: distance visual acuity
eCSC: effective cataract surgical coverage
eREC: effective refractive error coverage
MSVI: moderate-severe visual impairment
RAAB: Rapid Assessment of Avoidable Blindness
URE: uncorrected refractive error
VA: visual acuity
VI: visual impairment
WHO: World Health Organization
